# Modelling the Impact of Chronic Cigarette Smoke Exposure in Obese Mice: Metabolic, Pulmonary, Intestinal, and Cardiac Issues

**DOI:** 10.3390/nu12030827

**Published:** 2020-03-20

**Authors:** Emilie Dubois-Deruy, Gaëlle Rémy, Jeanne Alard, Gwenola Kervoaze, Maggy Chwastyniak, Morgane Baron, Delphine Beury, Léa Siegwald, Ségolène Caboche, David Hot, Philippe Gosset, Corinne Grangette, Florence Pinet, Isabelle Wolowczuk, Muriel Pichavant

**Affiliations:** 1 University of Lille, Inserm, CHU Lille, Institut Pasteur de Lille, U1167 - RID-AGE - Facteurs de risque et déterminants moléculaires des maladies liées au vieillissement, F-59000 Lille, France; emilie.deruy@pasteur-lille.fr (E.D.-D.); maggy.chwastyniak@pasteur-lille.fr (M.C.); florence.pinet@pasteur-lille.fr (F.P.); 2 University of Lille, CNRS UMR9017, Inserm U1019, CHRU Lille, Institut Pasteur de Lille, CIIL - Center for Infection and Immunity of Lille, 59000 Lille, France; gaelle.remy@pasteur-lille.fr (G.R.); jeanne.alard@gmail.com (J.A.); gwenola.kervoaze@pasteur-lille.fr (G.K.); morgane.baron@cnrs.fr (M.B.); delphine.beury@pasteur-lille.fr (D.B.); lea.siegwald@gmail.com (L.S.); segolene.caboche@pasteur-lille.fr (S.C.); david.hot@pasteur-lille.fr (D.H.); philippe.gosset@pasteur-lille.fr (P.G.); corinne.grangette@pasteur-lille.fr (C.G.); isabelle.wolowczuk@pasteur-lille.fr (I.W.)

**Keywords:** unhealthy lifestyle, cigarette, obesity, lung, heart, metabolism, gut, microbiota

## Abstract

Unhealthy lifestyle choices, such as bad eating behaviors and cigarette smoking, have major detrimental impacts on health. However, the inter-relations between obesity and smoking are still not fully understood. We thus developed an experimental model of high-fat diet-fed obese C57BL/6 male mice chronically exposed to cigarette smoke. Our study evaluated for the first time the resulting effects of the combined exposure to unhealthy diet and cigarette smoke on several metabolic, pulmonary, intestinal, and cardiac parameters. We showed that the chronic exposure to cigarette smoke modified the pattern of body fat distribution in favor of the visceral depots in obese mice, impaired the respiratory function, triggered pulmonary inflammation and emphysema, and was associated with gut microbiota dysbiosis, cardiac hypertrophy and myocardial fibrosis.

## 1. Introduction

Obesity and cigarette smoking are important risk factors for many age-related diseases and, as such, remain major public health challenges [1]. Worldwide obesity has more than doubled since the 1980s, and over a third of the world’s population is currently overweight or obese, including children and adolescents [2]. This is a serious concern since obesity is associated with poorer mental health outcomes and reduced quality of life, and represents the fifth leading cause of premature deaths due to, e.g., type 2 diabetes or heart diseases [3]. Besides, obesity is associated with lung dysfunction, lung inflammation and lung remodeling [4,5]. On the other hand, while being primarily associated with respiratory diseases, smoking also increases the risk of metabolic and cardiovascular diseases, and is the top one preventable cause of death in developed countries [6]. Importantly, obesity and cigarette smoking both predispose to chronic obstructive pulmonary disease (COPD) and modulate the progression of inflammatory bowel diseases (IBD) [7,8,9,10], partly through modulation of gut microbiota [11,12]. Moreover, cardiovascular diseases such as ischemic heart disease or heart failure are major complications in COPD patients [13,14] as well as in obese individuals [15]. Indeed, the Framingham study, which demonstrated the role play by cigarette smoking in the development of heart diseases, reported that the life expectancy of obese smokers is about 13 years shorter than the one of non-obese never smokers [16].

The inter-relation between obesity and cigarette smoking is complex and not fully understood, yet it likely involves inflammatory processes in metabolic, respiratory, intestinal, and cardiovascular tissues [5,17,18,19]. Several studies reported a higher incidence of metabolic syndrome in smokers [17], with a greater risk of developing type 2 diabetes [18] and cardiovascular diseases [19]. Smoking is generally associated with lower body weight, possibly by enhancing total energy expenditure [20]. However, active smokers who smoke more intensively tend to weigh more than light smokers. While this may be due to other lifestyle factors, such as physical inactivity and unhealthy diet consumption, it has been recently suggested that obesity influence smoking uptake and intensity [21].

Herein, we aimed at deciphering the joint associations of obesity and cigarette smoking at multiple organ level. We thus chronically exposed high-fat diet-fed obese mice to cigarette smoke and assessed metabolic, respiratory, intestinal, and cardiovascular functions. Altogether, the present work may bring clues on the health consequences of cigarette smoking in non-obese vs. obese individuals.

## 2. Materials and Methods

### 2.1. Objective and Design of the Study

**Figure 1:**
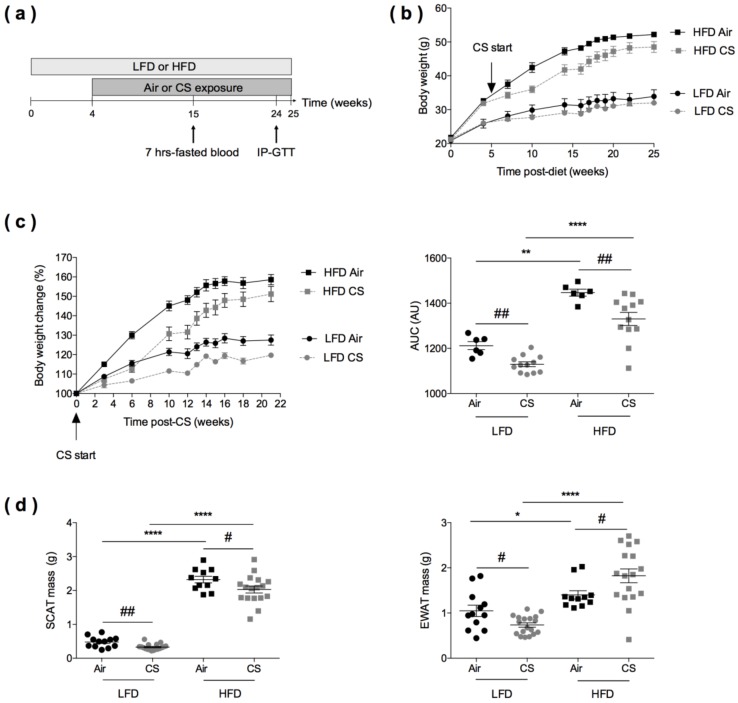
Effects of chronic cigarette smoke (CS) exposure on the body weight and fat mass of lean (LFD) and obese (HFD) mice. **(a)** Experimental design of the study: the day of the start of the study was defined as day 0 allowing the association of this time point with the schedule of CS in lean and obese mice. Mice were fed with low-fat diet (LFD, lean) or high-fat diet (HFD, obese) for four weeks and exposed to ambient air (Air) or to cigarette smoke (CS) (5 cigarettes/day, 5 days/week) for additional 21 weeks. Intraperitoneal glucose tolerance (IP-GTT) was performed at week 24. **(b)** Body weight evolution (expressed in g) of lean and obese mice exposed to Air (*n* = 6) or CS (*n* = 12) from week 0 to week 25. Data are expressed as mean ± SEM. **(c)** Body weight change from CS start to 21 weeks post-CS exposure (expressed as % body weight loss from body weight at CS start) (left panel), and corresponding area under the curve (AUC) values (expressed in arbitrary units (AU) (right panel). Data are expressed as mean ± SEM. **(d)** Masses (expressed in g) of subcutaneous (inguinal) white adipose tissue (SCAT) (left panel) and epididymal white adipose tissue (EWAT) (right panel) were recorded at sacrifice, *n* = 11–12 in Air groups and *n* = 17–18 in CS groups. Data are expressed as individual and mean ± SEM values. * *p* < 0.05, ** *p* < 0.01, **** *p* < 0.0001, * correspond to diet effect (HFD vs. LFD), and # *p* < 0.05, ## *p* < 0.01, #correspond to CS effect (CS vs. Air).

The objective of the study was to compare the metabolic, immunological and inflammatory consequences of the chronic exposure to cigarette smoke (CS) in lean and obese mice. Each experiment was performed at least in duplicate, including a number of mice per experimental group that allowed statistical analyses and adequate evaluation of the possible size effect, as depicted in Figure 1a.

### 2.2. Experimental Animal Model

Six to eight-week-old male C57BL/6JRj mice were purchased from Janvier (Le Genest-St-Isle, France) and housed in specific pathogen-free environment in Lille Pasteur Institute’s animal facilities (accredited no. C59-350009) and maintained in a temperature-controlled (20 ± 2 °C) environment with a strict 12-hours dark/light cycle. Housing and experimentations were carried out according to the French government guidelines of laboratory animal care and approved by the Departmental Direction of Veterinary Services (Prefecture of Lille, France), European guidelines of laboratory animal care (number 86/609/CEE) and French legislation (Government Act 87-848). The present project has been approved by the national Institutional Animal Care and Use Committee (CEEA 75) and received the authorization number APAFIS# 7281. Four weeks after the beginning of diets irradiated HFD (60% kcal fat; D12492) and LFD (10% kcal fat; D12450B) from Research Diets (Brogaarden, Lynge, Denmark), mice started to be exposed to cigarette smoke (CS) generated from 5 cigarettes per day (3R4F research cigarettes from the University of Kentucky, Lexington, KY, USA), 5 days a week and up to 21 weeks (Emka technologies, SCIREQ Inc., Montreal, Qc, Canada) [22]. Control groups were exposed to ambient air (Figure 1a). All measurements were taken from distinct samples. Individual body weights were weekly recorded.

### 2.3. In Vivo Magnetic Resonance Imaging (MRI)

All MRI examinations were performed on a 7T Bruker Biospec (Ettlingen, Germany) imaging system equipped with a 20 cm horizontal bore magnet as previously described [23]. Anesthesia was induced by 2% of isoflurane and maintained at 1%–1.5% along acquisition depending of respiration frequency. A pneumatic pillow (SA Inc. Stony Brook NY, USA) was used to monitor and perform respiratory triggering and gating. The mouse was placed in a cylindrical coil with a 39 mm inner diameter. Multi-slice echo gradient sequence was synchronized with respiration and performed to assess mouse position inside the magnet following sequence parameters: RT/ET (Repetition Time/Echo Time) = 200/3 ms, FA (Flip Angle) = 30 °.

Adipose tissue images were acquired by a respiratory-gated T1-weighted imaging using coronal RARE (Rapid Acquisition with Relaxation Enhancement) sequence which covered thorax and abdominal regions. Multislice coronal images (FOV = 7 × 7 cm, slice thickness = 1.5 mm) were collected with a RT of 400 ms, and a ET of 9 ms, a 256 × 256 data matrix, and a number of repetitions (NEX) of 2. Image-based 3D reconstructions were built for the evaluation of adipose tissue volumes. Reconstructions were manually drawn on ITKSnap software for each T1-weighted image sets.

### 2.4. Intra-Peritoneal Glucose Tolerance Test (IP-GTT)

IP-GTT was performed after 24 weeks of diet, as previously described [24]. Blood glucose levels were measured with an automatic glucometer (ACCU-CHEK Performa, Roche, Mannheim, Germany), before and 15, 30, 60, 120, and 180 min after glucose administration (1 g/kg, Sigma Aldrich, Lyon, France) on overnight fasted mice, by tail-tip bleeding.

### 2.5. Evaluation of Lung Function

Mice were anaesthetized, tracheotomized, and cannulated before being connected to a flexiVent FX system (SCIREQ Inc., Montreal, Qc, Canada) and operated by the flexiWare software v7.7. Immediately after connection to the ventilator, set at 150 breaths / min, two deep lung inflations were performed at least 6–12 seconds apart to recruit lung beyond any closed airway and to standardize lung volume history. This was done by inflating the lungs to 30 cm H_2_O over 3 seconds and holding that pressure for another 3 seconds to allow for the lungs to equilibrate after the inflation. Mice were then submitted to a 300 breaths/min hyperventilation in order to eliminate spontaneous breathing before a 150 breaths / min return. Next, the mechanical properties of the mouse respiratory system were assessed at baseline, i.e., before the construction of a full-range pressure–volume (PV) curve. This was done using a sequence of measurements integrated by default in the flexiVent operating software and referred to as the mouse mechanics scan [25]. The area between the PV loop inflation and deflation limbs (Hysteresis) was then calculated.

### 2.6. Assessment of Airway Inflammation

Bronchoalveolar lavages (BAL) and pulmonary cells were prepared and analyzed by flow cytometry as previously described [22]. BAL samples were obtained by washing the lungs twice with 1 ml of phosphate-buffered saline solution (PBS) plus 2% fetal bovine serum (FBS) (Gibco). After centrifugation at 400 g for 6 min at 4 °C, the supernatant (cell-free BAL fluid) was stored at −20 °C for cytokine analysis. The pellet was resuspended in PBS 2% FBS, total cell numbers per BAL were determined and used for flow cytometry analysis. Briefly, the left lobe of the lung was mashed with a sterile blade then digested with collagenase (1 mg/mL, Collagenase Type VI 17104–019 Gibco by Life technologies) at 37 °C. After 15 min of digestion, lungs were homogenized with an 18G needle and digested for 15 min. After centrifugation at 400 g for 6 min at 4 °C, the pellets were resuspended in a 30% Percoll solution (Percoll TM GE Healthcare 17–0891-01) and centrifuged at 500 g for 15 min. The pellets were resuspended in red blood cells (RBC) lysis buffer during 5 min at 20 °C, to remove erythrocytes. The reaction of RBC lysis was stopped with PBS 2% FBS (Gibco). After centrifugation at 400g for 6 min at 4 °C, pulmonary cells were resuspended in PBS 2% FBS, then enumerated and used for flow cytometry. Cells were acquired and analyzed on a Fortessa (Becton Dickinson, Rungis, France) cytometer, using the FlowJo software. Antibodies specific for mouse F4/80 (PE conjugated), CD45 (Vio-Green conjugated), were purchased from Miltenyi Biotech (Bergisch Gladbach, Germany). Antibodies specific for mouse CD11c (PE-Cy7 conjugated), CD86 (Alexa Fluor 700 conjugated), were purchased from BD Biosciences (Le Pont de Claix, France).

### 2.7. Cytokines and Insulin Quantification

CXCL1 and IL-6 concentrations were determined in lung protein extracts, blood leptin, and insulin levels (post a 7-hours-fasting period) were quantified by ELISA. The homeostatic model for assessment of the Insulin Resistance index (HOMA-IR) was calculated as ((fasted serum insulin × fasted serum glucose)/22, 5) [26].

### 2.8. Histological Analyses

To study lung remodeling, the inferior lobe of the lungs was inflated and fixed in formalin. Lungs were paraffin-embedded; cross-sections were cut and stained with hematoxylin and eosin as previously described [22,27]. Random areas were selected for disease scoring which include both lung remodeling and inflammation (Table S1). Mean linear intercept was measured as the mean length of straight line segments (“chords”) on random test lines spanning the air space between two sequential intersections of the alveolar surface with the test line.

Cardiac remodeling was quantified as previously described [28]. Transversal cardiac myocyte cryo-sections were defrosted slowly in acetone before being washed (PBS; 3 × 5 min). After a 2 h incubation in the dark with Rhodamine-conjugated wheat germ agglutinin (WGA) purchased from EUROBIO/ABCYS (Les Ulis, France) (dilution 1/150), sections were washed (PBS; 3 × 5 min), mounted with Vectashield and observed with LSM710 confocal microscope. The data analysis was performed with Axiovison software (Zeiss).

### 2.9. RNA Extraction and Quantitative RT-PCR

Ileal segments were stored in RNA later (Sigma, Saint-Louis, MI, USA) and the left ventricles of the heart were frozen at −80 °C.

Ileal samples were homogenized using Lysing Matrix D (MP Biomedicals, Eschwege, Germany) and total RNA was extracted using NucleoSpin RNAII isolation kit (Macherey-Nagel Duren, Germany) according to manufacturer’s recommendations. RNA (1 μg) was reverse transcribed using the High capacity cDNA reverse transcription kit (Applied Biosystems™, Foster City, CA, USA). RT-qPCR was performed using the Power SYBR Green PCR Master Mix on the QuantStudio™ 12K Flex Real-Time PCR System (Applied Biosystems, New Jersey, NJ, USA).

Total RNA from left ventricles was prepared using TRI Reagent (Sigma-Aldrich) according to manufacturer’s instructions. RNA (500 ng) was reverse transcribed with miScript II RT Kit (Qiagen). RT-qPCR was performed using the miScript SYBR Green PCR Kit (Qiagen). All samples were processed in duplicate reactions on a Stratagene Mx3005O (Agilent Technologies).

Primer sequences are available upon request. Relative mRNA levels (2^-Δ(ΔCt)^) were determined by comparing 1) the PCR cycle thresholds (Ct) for the gene of interest and the housekeeping gene *Actb* (ΔCt) for ileum and *Hprt* for heart and 2) ΔCt values for exposed and control groups (ΔΔCt).

### 2.10. Microbiota Analysis

Caecal samples were collected at sacrifice, immediately snap-frozen in liquid nitrogen and stored at −80 °C until use. DNA extractions were performed using the commercial bead beating method ZR Fecal DNA MiniPrep™ (ZR) (Zymo Research, Irvine, CA, USA). The quantity and the purity of DNA (expressed as the ratio of absorbance at 260 nm and 280 nm (*A*260/*A*280)) were assessed using a Nanodrop^®^ spectrophotometer.

For microbiota analysis, the sequencing library was generated by amplifying the V3-V4 region of the bacterial 16S-rRNA gene using 16S rRNA amplicon generation for MiSeq with the primers Bact-0341 (CCTACGGGNGGCWGCAG) and Bact-0785 (GACTACHVGGGTATCTAATCC). Individual samples were barcoded, pooled to construct the sequencing library and sequenced using an Illumina Miseq (Illumina, San Diego, CA) to generate paired-end 2x300 bp reads. Data was then normalized using the QIIME 1.9 script normalize_table.py (using CSS). Alpha diversity and beta diversity (weighted and unweighted UniFrac) analyses were performed with QIIME 1.9 scripts. Differential OTU abundance analyses between exposed and control groups were performed with STAMP v2.1.3 using the Welch’s test two-side and the Benjamini-Hochberg FDR correction.

### 2.11. Statistical Analysis

Data are expressed as means ± SEM and were analyzed with GraphPad software (GraphPad, San Diego, CA, USA). Data were compared using nonparametric Mann–Whitney test for 2 groups’ comparison. Statistical significance of CS effect compared to Air and/or HFD effect compared to LFD was accepted at a *p* value < 0.05.

## 3. Results

### 3.1. Experimental Design for Evaluating the Consequences of Chronic Cigarette Smoke Exposure in Lean and Obese Mice

The scheme of animal treatments is reported in Figure 1a. At the start of the study, mice were fed with low-fat diet (LFD) or high-fat diet (HFD) for four weeks, before being exposed to cigarette smoke (CS) (5 cigarettes per day, 5 days a week) or ambient air for 21 additional weeks. Body weights were weekly recorded, blood was taken from 7-hours-fasted mice at 15 weeks (11 weeks post-CS or Air exposure), in vivo tolerance to glucose (IP-GTT) was assessed at 24 weeks, and mice were sacrificed at 25 weeks.

### 3.2. Chronic Cigarette Smoke Exposure Induces White Adipose Tissue Redistribution in Obese Mice

Relative to LFD-fed animals, HFD-fed mice expectedly gained weight, both in the Air and CS conditions (Figure 1b). Importantly, chronic CS exposure significantly limited body weight gain in lean (LFD) and obese (HFD) animals (Figure 1c).

At the end of the protocol, the subcutaneous (inguinal) white adipose tissue (SCAT) and the visceral (epididymal) white adipose tissue (EWAT) were weighed (Figure 1d). It showed that both the SCAT and EWAT masses were lower in CS-exposed lean mice than in Air-exposed animals. When compared to controls, obese mice exposed to CS presented a lower SCAT mass and, strikingly, a higher EWAT mass. Thus, we further assessed the quantity and distribution of body fat tissue depots (SCAT, EWAT, and peri-renal) in lean and obese mice exposed to Air or CS, using magnetic resonance imaging (MRI) (Figure S1). It confirmed the decreased SCAT mass in CS exposed LFD mice, when compared to Air controls (*p* < 0.057). Despite not significant, there was a tendency for increased intra-abdominal fat (epididymal and peri-renal fat depots) in HFD mice upon CS exposure. Taken together, the results show that chronic CS exposure limits body weight gain in both lean and obese mice and specifically altered fat tissue redistribution (in favor of the visceral depot) in obese animals.

### 3.3. Chronic Cigarette Smoke Exposure Has No Impact on Glucose Homeostasis in Lean and Obese Mice

**Figure 2:**
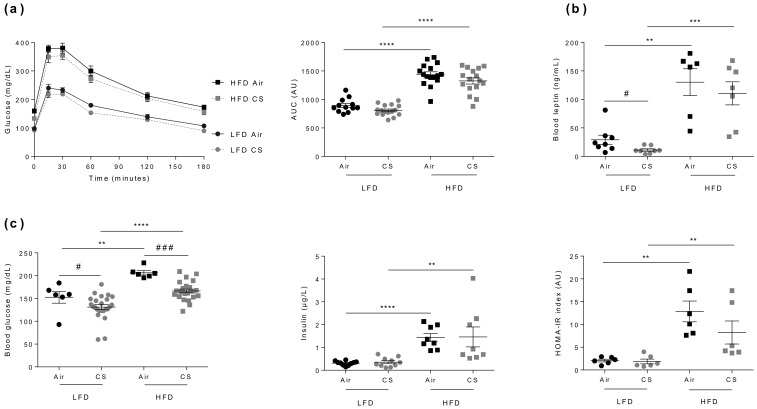
Chronic cigarette smoke (CS) exposure has no impact on glucose homeostasis in lean (LFD) and obese (HFD) mice. **(a)** IP-GTT was performed at week-24. Blood glucose levels (expressed in mg/dL) were measured in overnight-fasted mice (0) and at the indicated times following glucose administration (left panel). The corresponding AUC values are shown (expressed in AU) (right panel), *n* = 12–16 per group. **(b)** Fasted (7 hours fasting) blood leptin levels (expressed in ng/mL) were quantified at week-15 (*n* = 6–8 mice per group). **(c)** Fasted (7 hours fasting) blood glucose (expressed in mg/dL) (left panel) and insulin (expressed in µg/L) (middle panel) levels were evaluated at week-15, and the homeostatic model for assessment of insulin resistance (HOMA-IR) was calculated (right panel), *n* = 6 mice per group. Data are expressed as individual and mean ± SEM. values. ** *p* < 0.01, *** *p* < 0.001, **** *p* < 0.0001, * correspond to diet effect (HFD vs. LFD), and # *p* < 0.05, ### *p* < 0.001, #correspond to CS effect (CS vs. Air).

Obesity is often associated with glucose intolerance, elevated leptin, glucose and insulin blood levels, and insulin resistance [29]. Besides, CS was proposed to alter glucose homeostasis and to cause insulin resistance [30], yet this allegation still remains debated [31]. As expected, compared to the respective lean controls, Air- or CS-exposed obese mice were significantly glucose intolerant (Figure 2a), had higher blood leptin levels (Figure 2b), and were insulin resistant (as estimated by HOMA-IR index calculation) (Figure 2c).

In accordance with CS-exposure-induced body weight loss (see Figure 1c), blood leptin levels were lower in CS-exposed animals (Figure 2b). However, CS exposure had no significant impact on tolerance to glucose, neither in lean nor in obese mice (Figure 2a), despite a significant CS-induced decrease in fasted blood glucose levels in both animal groups (Figure 2c). Altogether, we show that chronic CS exposure has no major impact on glucose homeostasis in lean or obese mice.

### 3.4. Lung Dysfunction and Inflammation Induced by Chronic Cigarette Smoke Exposure are Worsened in Obese Mice

**Figure 3:**
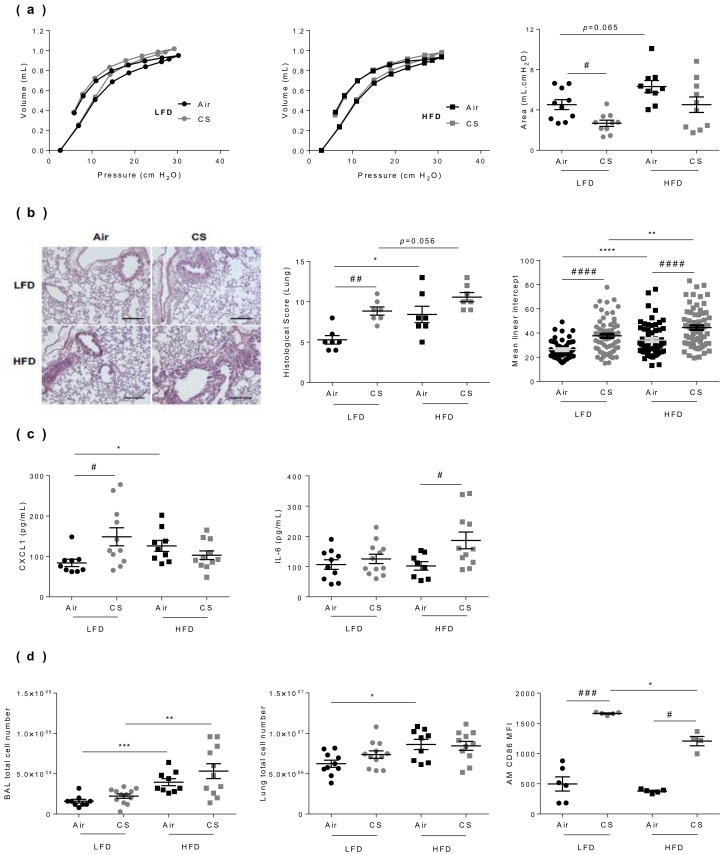
Lung dysfunction and inflammation induced by chronic cigarette smoke (CS) exposure are worsened in obese mice. **(a)** The pressure-volume (PV) loops were determined by the flexiVent FX system in lean (LFD, left panel) and obese (HFD, middle panel) mice, and the areas between inflating and deflating curves (right panel) were calculated (mL.cm H_2_O) (*n* = 10–12 mice per group). **(b)** Hematoxylin-eosin-stained lung sections (left panel) were used for calculation of the histological score (middle panel) (*n* = 7 per group) (see Table S1 for studied parameters), and mean linear intercept (right panel) (*n* = 53–88). **(c)** Concentrations of CXCL-1 and IL-6 in lung protein extracts by ELISA (pg/mL) (*n* = 9–12 mice per group). **(d)** Total cell counts in broncho-alveolar lavage (BAL) (left panel) and lung tissue (middle panel), and mean fluorescence intensity (MFI) of CD86-expressing CD45^+^ F4-80^+^ CD11c^+^ alveolar macrophages (AM) (right panel) determined by flow cytometry (*n* = 9–12 mice per group). Data are expressed as individual and mean ± SEM values. * *p* < 0.05, ** *p* < 0.01, *** *p* < 0.001, **** *p* < 0.0001, * correspond to diet effect (HFD vs. LFD), # *p* < 0.05, ## *p* < 0.01, ### *p* < 0.001, #### *p* < 0.0001, #correspond to CS effect (CS vs. Air).

We then compared the impact of CS exposure on the respiratory function of lean and obese mice, using an invasive approach (Figure 3a). As we previously reported, both chronic CS exposure [22] and obesity [5] separately altered lung function in mice. CS exposure modulated the pressure–volume (PV) loop in lean mice, but not significantly in obese mice (Figure 3a). No cumulative effect of CS and HFD was observed on the alteration of the lung function. Taken independently, both regimen and CS exposure have significant effects on tissue morphometry (Figure 3b). Obesity itself triggered some tissue lesions, as well as CS exposure alone. In addition, histopathological lung scoring revealed that obesity and CS exposure exerted cumulative deleterious effects, in terms of tissue lesions. Indeed, CS exposure increased emphysema both in lean and obese mice, as shown by the quantification of the mean linear intercept using a morphometric method. This lung structural alteration was even majored when obese mice were exposed to CS compared to controls (Figure 3b and Table S1).

To further characterize CS exposure-induced changes in lean vs. obese mice, we next quantified inflammatory cytokines levels in lung protein extracts. When compared to respective controls, CS exposure led to increase CXCL-1 levels in lean mice and IL-6 levels in obese mice (Figure 3c), while IL-1β concentration was not impacted in lung tissues (data not shown). We then evaluated inflammation through enumerating cells in the bronchoalveolar lavage (BAL) and in the lung parenchyma (Figure 3d). It showed that obesity increased BAL and lung cell numbers, and that chronic CS exposure enhanced the obesity-induced increase in BAL cells. In addition, while obesity had no impact on CD45^+^ F4/80^+^ CD11c^+^ alveolar macrophage number, chronic CS exposure increased their frequenciesas previously described [22], and their activation status as depicted by the increased expression of CD86, in both lean and obese animals.

### 3.5. Chronic Cigarette Smoke Exposure Limits Obesity-Associated Gut Inflammation, Exacerbates Fatty Acid Metabolism and Modulates Caecal Microbiota

**Figure 4:**
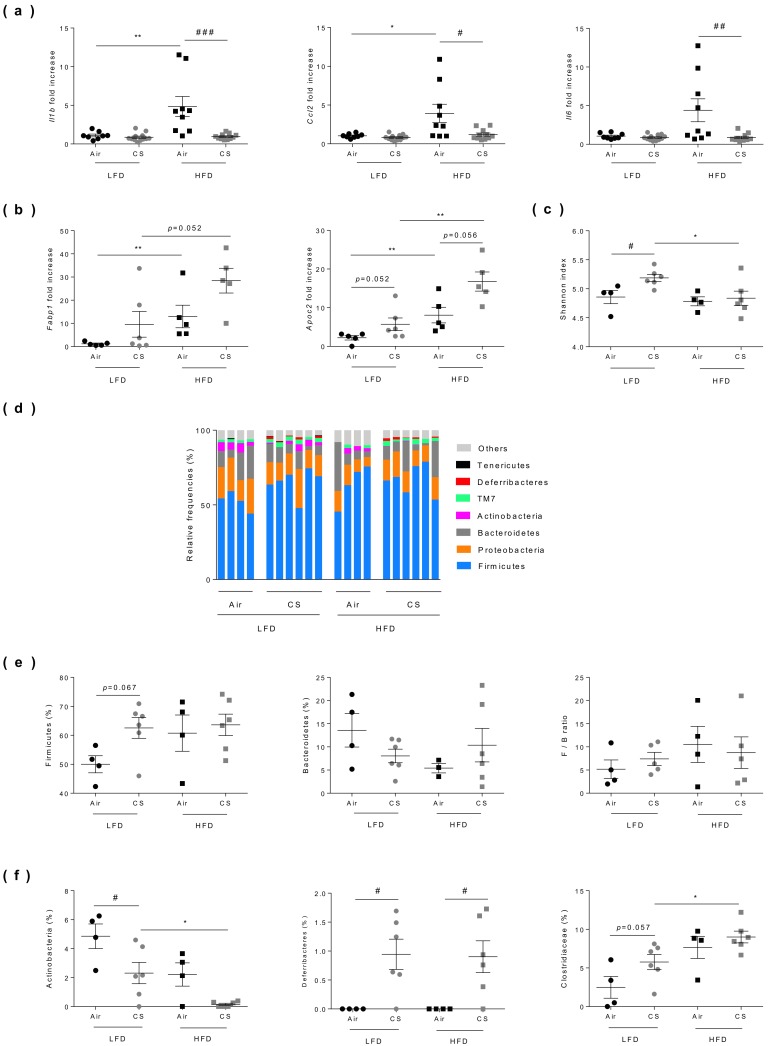
Chronic cigarette smoke (CS) exposure limits obesity-associated gut inflammation, exacerbates fatty acid metabolism and modulates caecal microbiota. **(a)** The expression levels of Il1b, Il6, and ccl2 genes evaluated in ileum by RT-qPCR (*n* = 8–12 mice per group). **(b)** The expression levels of Fabp1 and Apoc2 genes evaluated in ileum by RT-qPCR (*n* = 5–6 mice per group). **(c)** Shannon diversity index was determined (*n* = 4–6 mice per group). (**d**) Relative abundance of bacterial 16S rRNA genes found in caecal content classified at phyla level. Individual profiles are shown for each group of mice (*n* = 4–6 mice per group). **(e)** Relative frequencies of Firmicutes, Bacteroidetes and Firmicutes/bacteroidetes ratio found in caecal contents. (**f**) Abundance of Actinobacteria (left panel), Deferribacteres (middle panel) and Clostridiaceae (right panel) found in caecal contents. Data are expressed as individual and mean ± SEM values. * *p* < 0.05, ** *p* < 0.01, *correspond to diet effect (HFD vs. LFD), # *p* < 0.05, ## *p* < 0.01, ### *p* < 0.001, #correspond to CS effect (CS vs. Air). See Table S2 for detailed informations.

Cigarette smoking and obesity have both been reported to alter the composition and diversity of the gut microbiota, and to be associated with gut inflammation [32,33,34]. We thus compared the consequences of chronic exposure to CS on gut inflammation and microbiota composition in lean and obese mice (Figure 4). As expected, obesity was associated to increased inflammation of the ileum, as revealed by the significantly higher mRNA expression level of Il1b, Ccl2 and, to a lesser extent, of Il6. Chronic exposure to CS had no impact on Il1b, Il6, and Ccl2 expression levels in lean mice whereas it significantly reduced the expression of all these pro-inflammatory genes in obese mice (Figure 4a).

The proteins involved in fatty acid (FA) binding (such as FABP-1) and metabolism (such as Apolipoprotein C2) are also tightly regulated at the gut level, facilitating the sensing of dietary fat. Compared to lean mice, expression levels of the genes encoding these proteins were significantly increased in the small intestine of obese mice, and interestingly they were even overexpressed after CS exposure (Figure 4b).

Compared to lean animals, no change in bacterial diversity (Shannon index) was observed in obese mice, while chronic CS exposure significantly enhanced bacterial diversity in lean mice only (Figure 4c). Although the observed differences were not statistically significant, we confirmed, as previously reported in the literature [35], that obesity altered the composition of gut microbiota (Figure 4d) notably by increasing the proportion of Firmicutes while decreasing the levels of Bacteroidetes. However, the Firmicutes/Bacteroidetes ratio was not significantly impacted by chronic CS exposure neither in lean nor in obese mice (Figure 4e and Table S2). Nevertheless, a significant drop (50% decrease) of Actinobacteria (and more particularly Bifidobacteria) was observed in lean mice exposed to CS, while these bacteria totally disappeared in obese-CS exposed mice (Figure 4f and Table S2). We also observed a significant increased level in the Deferribacteres phylum in all CS-exposed mice and an increase in Clostridiaceae in obese mice (Figure 4f and Table S2).

### 3.6. Chronic Cigarette Smoke Exposure Restricts Obesity-Induced Cardiac Hypertrophy

**Figure 5:**
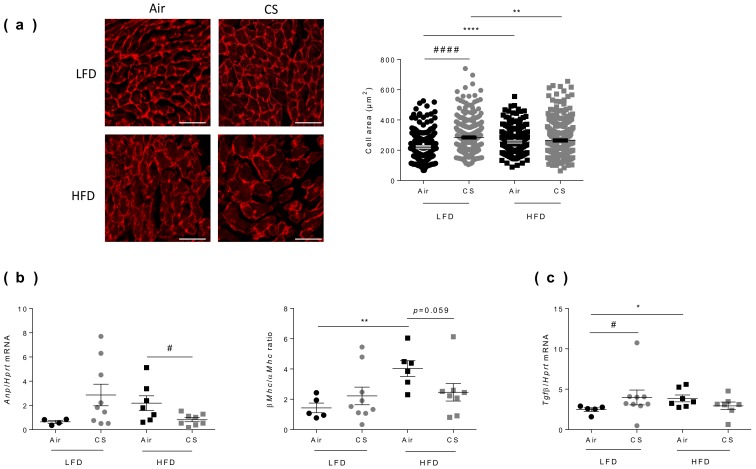
Chronic cigarette smoke (CS) exposure restricts obesity-induced cardiac hypertrophy. **(a)** Cardiomyocytes transverse section areas were stained by rhodamin labeled-wheat germ agglutinin (WGA) (left panel) and cells’ area were quantified (right panel) (*n* = 5–8 mice per group; 224-346 cells per group). Scale bar corresponds to 50 µm. **(b)** Fetal gene program expression of Atrial Natriuretic Peptide (Anp) and β/α Myosin Heavy Chain (Mhc) ratio were quantified by RT-qPCR (*n* = 4–9 mice per group). **(c)** Fibrosis was measured by quantification of transforming growth factor beta (Tgfβ) expression by RT-qPCR (*n* = 6–9 mice per group. Data are expressed as individual and mean ± SEM. values. * *p* < 0.05, ** *p* < 0.01, **** *p* < 0.0001, *correspond to diet effect (HFD vs. LFD), # *p* < 0.05, #### *p* < 0.0001, #correspond to CS effect (CS vs. Air).

Cardiac hypertrophy corresponds to the abnormal enlargement of the heart, resulting from increased cardiomyocyte size and changes in other heart muscle components, such as the extracellular matrix [36]. To compare the effect of chronic CS exposure on cardiac hypertrophy in lean and obese mice, we evaluated the morphology of the left ventricle by measuring cardiomyocyte areas using fluorescence microscopy after wheat germ agglutinin (WGA) staining (Figure 5a). As expected from literature, obesity was associated with a significant increase in cardiomyocyte areas. Interestingly, chronic CS exposure led to cardiomyocyte enlargement in lean mice only (Figure 5a).

In response to mechanical or neurohormonal stimuli, the heart adapts to increased workload by changes in gene expression, particularly by reactivating some fetal genetic programs [37]. The most common studied genes are the cardiac myosin isoform [38] and the atrial natriuretic peptide (Anp) [39,40]. CS exposure tended to increase the expression of fetal genes encoding for Anp and β/α Myosin Heavy Chain ratio (β/αMhc) in lean mice (Figure 5b). Obesity per se significantly increased the β/αMhc ratio (Figure 5b). CS exposure in obese mice significantly decreased the expression of Anp and decreased the β/αMhc ratio. These results may partially result from the decreased body weight of CS-exposed obese mice (see Figure 1a). Indeed, the heart weight and cardiac hypertrophy have been reported to positively correlated to body weight [28].

Another hallmark of cardiac remodeling is cardiac fibrosis, which is characterized by an increase in collagens and other extracellular matrix components in the interstitium and perivascular regions of the myocardium and highly controlled by transforming growth factor beta (TGFβ) [41]. Interestingly, CS exposure significantly increased Tgfβ expression in lean mice. Obesity was also associated to enhanced Tgfβ expression, which remained unchanged upon CS exposure (Figure 5c).

## 4. Discussion

Strong evidence shows that tobacco use and unhealthy diets increase the risk of many adverse conditions, including the world’s major non-communicable chronic diseases (NCD) such as chronic respiratory diseases, IBD, type 2 diabetes and cardiovascular diseases [42]. Although smoking and obesity are public health priorities in industrialized countries, the overlap between the two conditions has scarcely been investigated [13,15]. In the present study, we established a model of chronic co-exposure to cigarette smoke in obese and lean mice and evaluated the consequence at different organ level (lung, adipose tissues, gut, and heart), aiming at better deciphering the comorbidities [43] associated to unhealthy lifestyle in order to propose innovative strategies to prevent and/or treat NCD.

As observed in humans, we and others previously reported in experimental models that HFD-induced obesity leads to metabolic abnormalities such as insulin-resistance and glucose intolerance; increasing the risk for progressing to type 2 diabetes [24,44]. Whether CS may favor the development of metabolic disorders is still a matter of debate [45]. However, CS is often associated with insulin resistance, dyslipidemia and systemic inflammation, and epidemiological studies suggest an increased risk for diabetes associated with smoking [17,46,47]. As described in humans [45], we showed that CS exposure limited obesity-induced body weight gain. Intriguingly, despite lower body weights, CS mice presented increased visceral fat mass, mimicking the situation observed in humans [48]. It is known that excessive amounts of visceral fat have deleterious metabolic consequences [49]. Therefore, the body fat redistribution in favor of visceral depots evidenced in CS-mice may participate to the impaired glucose and lipid metabolism associated with smoking. However, we did not observe an impact on the development of glucose intolerance. The mechanisms that regulate body fat distribution are still incompletely known [50]. In that sense, the deeper analysis of fat redistribution in response to CS in our experimental model, might lead to the identification of novel cellular and molecular factors involved in this process.

Obesity or CS are independently associated with pulmonary inflammation and impaired lung function, both in humans [51] and in mice [4,5,22]. In our model, we confirmed that CS alters lung function and induces lung inflammation. Additive and perhaps synergistic effects of smoking and obesity could be observed on pulmonary outcomes, including lung remodeling and most of the inflammatory parameters that have been evaluated. However, we also demonstrated that obesity could take over CS-induced lung dysfunctions. This is in discordance with the additive effects of smoking and obesity on bronchial hyper-responsiveness observed by Sposato et al. [52]. In addition, we showed that CD86 expression is significantly lower on macrophages from CS-obese mice than from CS-lean mice. There are several evidences in literature showing that CD86 expression can be decreased (even suppressed) upon chronic stimulation due to increased IL-10 production, and is not further inducible following additional stimulus. This could be the case in our study since IL-10 levels are known to be increased in obesity conditions [53,54,55,56,57,58]. Therefore, the autocrine HFD-induced IL-10 production could limit CD86 expression on macrophages in response to CS.

Cigarette smoke has also been reported to induce gut inflammation [5,59], however its impact on IBD-related gut inflammation can either be protective (e.g., ulcerative colitis) or detrimental (e.g., Crohn’s disease). Smoking also alters the composition and diversity of the gut microbiota (known as dysbiosis), but the impact of cigarette smoking on gut inflammation have not yet been clarified [11]. On the other hand, obesity is also associated to gut inflammation, gut permeability, and gut microbiota dysbiosis [32,33], and predisposes to the development of Crohn’s disease [9].

In our model, we confirmed that obesity is associated with intestinal inflammation. Surprisingly, chronic CS exposure neither induced gut inflammation in lean mice nor had additive (or synergistic) effect on obesity-induced intestinal inflammation. Rather, we found that CS decreased obesity-induced gut inflammation, likely resulting from CS-induced decrease in body weight.

As reported in humans, we showed higher expression of genes involved in fatty acid binding (*Fabp1*) and metabolism (*Apoc2*) in the intestine of obese mice than in lean controls. Interestingly, chronic CS exposure also enhanced *Fapb1* and *Apoc2* expression in the gut of lean mice and had additive effect on obesity-induced *Fabp*1 and *Apoc2* expression. The exact mechanism for *Apoc2* gene regulation in the gut remains unknown [60], but the intestinal fatty acid binding protein (I-FABP), which is abundantly present in enterocytes, has proven to be a useful marker for small intestine mucosal damage [61].

Regarding the impact of obesity and CS on gut microbiota, we showed that obesity was associated with gut dysbiosis, notably a change in the Bacteroidetes-to-Firmicutes ratio and a drop in Bifidobacteria, as already reported [44,62]. Interestingly, chronic CS exposure differentially impacted the level of Bacteroidetes in lean vs. obese mice and exacerbated the obesity-induced decrease in Bifidobacteria levels. These observations are of importance since Bifidobacteria are well known to exhibit health-promoting abilities, notably in metabolic diseases [44]. It has to be noted that a recent review reported that the intestinal microbiome is altered by smoking notably with decreased levels of Actinobacteria as well as the genera Bifidobacteria [11]. Therefore, we propose that the health detrimental effects resulting from the co-exposure to CS and obesity may partly rely upon the decreased abundance of these beneficial bacteria. However, since gut inflammation was decreased in co-exposed mice, we could suggest the emergence of a yet-to-be-identified anti-inflammatory microbial component. Interestingly, we observed a significant increase of Defferibacteres in all CS-exposed mice. It has been recently reported an increase of these bacteria, especially *Mucispirillum schaedleri*, the sole known representative of Deferribacteres present in the mammalian microbiota, in mice protected from *Salmonella typhimurium*. This bacteria could compete for nutrients, especially for nitrate and beneficially impacted on *S. thyphimurium*-induced colitis [63]. This mucus-associated bacterium is adapted to the high-redox environment of the mucus layer and is well equipped to handle oxidative bursts that occur during inflammation. However, since it is often associated to inflammation, it remains important to define if this commensal plays a role in disease progression or protection [64].

CS and obesity are well-established independent risk factors for cardiovascular diseases such as myocardial infarction, stroke and heart failure [46,65]. Moreover, it was already described that HFD (meaning a diet containing 5.24 kcal/g, 60 kcal% fat, 20 kcal% carbohydrate, and 20 kcal% protein) [66] as well as CS exposure [65] induced cardiac hypertrophy and fibrosis. Here, we confirmed that obesity or CS independently induce marked heart hypertrophic remodeling. These results are in accordance with a transcriptomic study described by Tilton et al., revealing an extensive cardiac response to CS in lean mice, in striking contrast with obese mice [67]. Interestingly, we also observed that obesity, as already shown by Wang et al. [68], as well CS exposure significantly increased cardiac fibrosis characterized by *Tgfb* mRNA expression but only in lean mice.

## 5. Conclusions

In conclusion, we herein reported for the first time the comparative analysis of the impact of chronic cigarette smoke exposure in obese vs. lean mice, at the lung, adipose tissue, gut, and intestine level. Our model allowed to mimic most of the features that have been reported in humans co-exposed to high-fat diet and cigarette smoke. However, the absence of additive or synergistic effects of cigarette smoke exposure on obesity-induced alterations might be due to the highly lipid-enriched (60%) diet that we used, which is above the 30%–40% dietary fat brought by obesogenic western diets [69]. In addition, mice were exposed to 5 cigarettes per day, 5 days per week, a dose which might have been insufficient to modulate the effects induced by obesity. Thus, our model could be improved by using a lower-lipid enriched diet and/or by increasing the frequency of cigarette smoke exposure. Despite its limitations, the present model may bring important clues on the health consequences of cigarette smoking in non-obese vs. obese individuals at pulmonary, cardiovascular, intestinal, and metabolic levels. Its translational value could be further enhanced when addressing different types of diets (obesogenic and/or diabetogenic). Ultimately, it could lead to the identification of novel therapeutic strategies that shall help the practitioners to clinically treat the adverse health effects associated with smoking in overweight and obese individuals. Such multidisciplinary approaches to medicine should lead to personalized clinical applications for treating NCD in the future and, thereby, improve global health.

## Supplementary Materials

The following are available online at https://www.mdpi.com/2072-6643/12/3/827/s1, Figure S1: Fat depot distribution in lean and obese mice chronically exposed to cigarette smoke, Table S1: Disease scoring of lung remodelling. Table S2: Relative abundance of caecal bacteria in lean (LFD) and obese (HFD) mice exposed or not (Air) to cigarette smoke (CS).
