# Erratum: Rémy et al. Modelling the Impact of Chronic Cigarette Smoke Exposure in Obese Mice: Metabolic, Pulmonary, Intestinal, and Cardiac Issues. *Nutrients* 2020, *12*, 827

**DOI:** 10.3390/nu13093084

**Published:** 2021-09-02

**Authors:** Gaëlle Rémy, Emilie Dubois-Deruy, Jeanne Alard, Gwenola Kervoaze, Maggy Chwastyniak, Morgane Baron, Delphine Beury, Léa Siegwald, Ségolène Caboche, David Hot, Philippe Gosset, Corinne Grangette, Florence Pinet, Isabelle Wolowczuk, Muriel Pichavant

**Affiliations:** 1 CIIL-Center for Infection and Immunity of Lille, Institut Pasteur de Lille, University of Lille, CNRS UMR9017, Inserm U1019, CHRU Lille, F-59000 Lille, France; remygaelle@aol.fr (G.R.); jeanne.alard@gmail.com (J.A.); gwenola.kervoaze@pasteur-lille.fr (G.K.); morgane.baron@cnrs.fr (M.B.); delphine.beury@pasteur-lille.fr (D.B.); lea.siegwald@gmail.com (L.S.); segolene.caboche@pasteur-lille.fr (S.C.); david.hot@pasteur-lille.fr (D.H.); philippe.gosset@pasteur-lille.fr (P.G.); corinne.grangette@pasteur-lille.fr (C.G.); isabelle.wolowczuk@pasteur-lille.fr (I.W.); 2 Inserm, CHU Lille, Institut Pasteur de Lille, University of Lille, U1167-RID-AGE-Facteurs de Risque et Déterminants Moléculaires des Maladies liées au Vieillissement, F-59000 Lille, France; emilie.deruy@pasteur-lille.fr (E.D.-D.); maggy.chwastyniak@pasteur-lille.fr (M.C.); florence.pinet@pasteur-lille.fr (F.P.)

The authors have requested that the following changes be made to their paper [1]. Due to a misinterpretation, the place of Dr Gaëlle Rémy was mistakenly under evaluated in the original published version. The original authors wish to add Dr. Gaëlle Rémy as the first coauthor to this paper. Contributions by Dr. Gaëlle Rémy included study methodology, formal analysis and validation, writing original draft preparation of the manuscript. We would like to apologize for any inconvenience caused to the authors and readers by this mistake. The published version will be updated on the article webpage, with reference to this notice.
