# Supplementary material for: Modelling the Impact of Chronic Cigarette Smoke Exposure in Obese Mice: Metabolic, Pulmonary, Intestinal, and Cardiac Issues
Source: Nutrients. 2020 Mar 20;12(3):827. doi: 10.3390/nu12030827 (PMC7175208; doi:10.3390/nu12030827)
Supplement: Supplementary file 1 [file nutrients-12-00827-s001.pdf]

SUPPLEMENTARY MATERIALS

Modelling the impact of chronic cigarette smoke exposure in obese mice: metabolic, pulmonary, intestinal, and cardiac issues

Emilie Dubois-Deruy[1]#, Gaëlle Rémy[2]#, Jeanne Alard[2], Gwenola Kervoaze[2], Maggy Chwastyniak[1], Morgane Baron[2], Delphine Beury[2], Léa Siegwald[2], Ségolène Caboche[2], David Hot[2], Philippe Gosset[2], Corinne Grangette[2], Florence Pinet[1], Isabelle Wolowczuk[2] and Muriel Pichavant[2]*

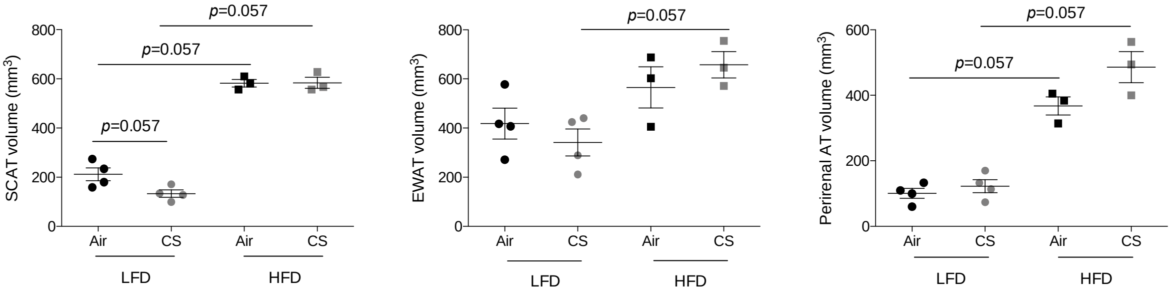

Figure S1: Fat depot distribution in lean and obese mice chronically exposed to cigarette smoke. Magnetic resonance imaging (MRI) was used to draw longitudinal slices of lean (LFD) and obese (HFD) mice exposed to Air or to CS. The manual labeling of the different fat depots was performed using the segmentation tool ITK-SNAP, and SCAT, EWAT and peri-renal adipose tissue volumes were calculated using the implementation of ITK-SNAP (n = 3-4 mice per group). Data are expressed as individual and mean ± SEM values.

Table S1: Disease scoring of lung remodeling.

**Lesion distribution**	No Lesion	1 to 25% of parenchyma	26 to 50% of parenchyma	51 to 75% of parenchyma	> 75% of parenchyma
Score	0	1	2	3	4
**Alveolar wall thickness**	< 1 erythrocyte	1 to 2 erythrocytes	3 to 5 erythrocytes	6 to 10 erythrocytes	> 10 erythrocytes
Score	0	1	2	3	4
**Hyaline membrane**	no			yes	
Score	0			1	
**Alveolar inflammation (PNN)**	Absent	< 10 / HP field	10 to 20 / HP field	21 to 50 / HP field	> 50 / HP field
Score	0	1	2	3	4
**Alveolar suppuration**	no			yes	
Score	0			1	
**Bronchiolar epithelium necrosis**	no			yes	
Score	0			1	
**Peribroncholiar**	Absent	< 10 / HP	10 to 20 /	21 to 50 /	> 50 / HP

**inflammation, PNN**		field	HP field	HP field	field
Score	0	1	2	3	4
**Peribronchiolar inflammation, mononuclear cells**	Absent	< 10 / HP field	10 to 20 / HP field	21 to 50 / HP field	> 50 / HP field
Score	0	1	2	3	4
**Vasculitis**	no			yes	
Score	0			1	
**Fibrinoid necrosis of vascular wall**	no			yes	
Score	0			1	

Randomly selected areas of lung sections were analyzed (see Figure S2a).
The score calculated from the distribution and type of lesions is highlighted in grey. HP: high power (400x).

Table S2: Relative abundance of caecal bacteria in lean (LFD) and obese (HFD) mice exposed or not (Air) to cigarette smoke (CS).

Taxonomy	LFD			HFD			*P* value
	Air	CS		Air	CS		
***Actinobacteria* phylum**	**4.86 ± 0.85[a]**	**2.32 ±0.74[a,b]**		2.21 ±0.80	**0.16 ±0.07[b]**		0.038[a], 0.028[b]
Coriobacteriaceae Family	0.00 ±0.00	0.00 ±0.00		**1.86 ±0.63**	**0.00 ±0.00**		0.033
Bifidobacteriaceae Family							
Bifidobacterium Genus	**4.70 ±0.87[a]**	**2.22 ±0.68[b]**		**0.38 ±0.22[a]**	**0.00 ±0.00[b]**		0.029[a], 0.015[b]
***Bacteroidetes* phylum**	14.25 ±3.80	8.40 ±1.54		12.44 ±6.78	10.84 ±3.77		
***Deferribacteres* phylum**	**0.00 ±0.00[a]**	**0.94 ±0.26[a,c]**		**0.00 ±0.00[b]**	**0.90 ±0.28[b]**		0.038[a], 0.038[b], 0.009[c]
***Firmicutes* phylum**	52.43 ±3.08	**65.14 ±3.69[a]**		63.89 ±6.75	66.75 ±4.01		0.041[a]
Clostridia Class	26.17 ± 2.02	33.25 ±2.46		37.81 ±0.05	42.28 ±2.52		
Ruminococcaceae Family	4.40 ±0.52	**4.16 ±0.35[a]**		3.51 ±0.28	4.70 ±0.87		0.002[a]
Clostridiaceae Family	2.49 ±1.41	**5.77 ±0.98[a, b]**		7.66 ±1.43	**9.01 ±0.76[b]**		0.002[a], 0.026[b]
Peptostreptococcaceae Family	**1.10 ±0.70[a]**	**4.34 ±0.46[a, b, c]**		5.09 ±0.74	**6.06 ±0.38[c]**		0.019[a], 0.004[b], 0.015[c]
***Proteobacteria* phylum**	20.07 ±2.11	**15.46 ±2.01[a]**		10.61 ±1.90	13.60 ±1.02		0.004[a]
Deltaproteobacteria Class							
Desulfovibrionaceae Family	**6.42 ±0.86[a]**	**4.18 ±0.20[a, b]**		3.79 ±0.73	4.47 ±0.35		0.019[a], 0.002[b]
Desulfovibrio genus	**3.71 ±0.34[a, d]**	**2.70 ±0.06[a, c, e]**		**0.00 ±0.00[b, d]**	**1.62 ±0.23[b, e]**		0.016[a], 0.010[b], 0.004[c], 0.029[d], 0.009[e]
***Tenericutes* phylum**	0.16 ±0.16	0.19 ±0.09	0.21 ±0.15	0.00 ±0.00	0.00 ±0.00	0.00 ±0.00	
***TM7* phylum**	**1.87 ±0.13[a]**	**2.76 ±0.21[a, b]**		1.09 ±0.63	2.59 ±0.40		0.010[a], 0.041[b]
***Unclassified* phylum**	4.74 ±0.48	**3.98 ±0.16**		5.10 ±0.30	**4.87 ±0.29**	4.96 ±0.31	0.015

Data are expressed as mean ± SEM values. Data significantly different between groups are marked in bold. *P* values are only indicated for significantly different data with an uppercase letter corresponding to the group analyzed.
